# Reading the epigenetic code for exchanging DNA

**DOI:** 10.7554/eLife.61820

**Published:** 2020-09-16

**Authors:** Mathilde Biot, Bernard de Massy

**Affiliations:** 1 Institut de Génétique Humaine, University MontpellierMontpellierFrance; 2 Centre National de la Recherche Scientifique, University MontpellierMontpellierFrance

**Keywords:** meiosis, recombination, double-strand breaks, histone modification, ZCWPW1, sexual reproduction, Human, Mouse

## Abstract

Three independent studies show that a protein called ZCWPW1 is able to recognize the histone modifications that initiate the recombination of genetic information during meiosis.

**Related research article** Mahgoub M, Paiano J, Bruno M, Wu W, Pathuri S, Zhang X, Ralls S, Cheng X, Nussenzweig A, Macfarlan TS. 2020. Dual histone methyl reader ZCWPW1 facilitates repair of meiotic double strand breaks in male mice. *eLife* **9**:e53360. doi: 10.7554/eLife.53360

**Related research article** Huang T, Yuan S, Gao L, Li M, Yu X, Zhang J, Yin Y, Liu C, Zhang C, Lu G, Li W, Liu J, Chen ZJ, Liu H. 2020. The histone modification reader ZCWPW1 links histone methylation to PRDM9-induced double-strand break repair. *eLife* **9**:e53459. doi: 10.7554/eLife.53459

**Related research article** Wells D, Bitoun E, Moralli D, Zhang G, Hinch A, Jankowska J, Donnelly P, Green C, Myers SR. 2020. ZCWPW1 is recruited to recombination hotspots by PRDM9, and is essential for meiotic double strand break repair. *eLife* **9**:e53392. doi: 10.7554/eLife.53392

Sexual reproduction relies on two sex cells, such as the egg and sperm, fusing together to form an embryo which contains two sets of chromosomes that carry a maternal and a paternal copy of the genome. Sex cells must therefore contain half the amount of genetic information as other cells in an organism. This is achieved through a specialized type of cell division called meiosis, which produces daughter cells that contain half as many chromosomes as their parent cell. Before the cell undergoes meiotic division, each chromosome exchanges genetic information with its copy via a process called homologous recombination (Hunter, 2015). This generates sex cells that are genetically unique, resulting in offspring that are distinct from each other and their parents.

Homologous recombination typically takes place at designated sites along the chromosome (de Massy, 2013). For instance, in primates and mice, recombination only occurs at locations where a protein called PRDM9 has bound to specific DNA motifs it recognizes (Grey et al., 2018). At these locations, PRDM9 modifies one of the histones (histone H3) that package DNA into chromatin by adding a methyl group to two of its amino acid residues (lysine 4 and lysine 36): this creates an epigenetic mark that provides an additional layer of information (on top of the genetic code) that can be read by certain proteins (Figure 1). For a protein to read the epigenetic marks deposited by PRDM9, it must be present in cells undergoing meiosis and contain domains that can bind to both modified sites in the histone. However, a protein that meets these requirements had not yet been identified.

**Figure 1.**
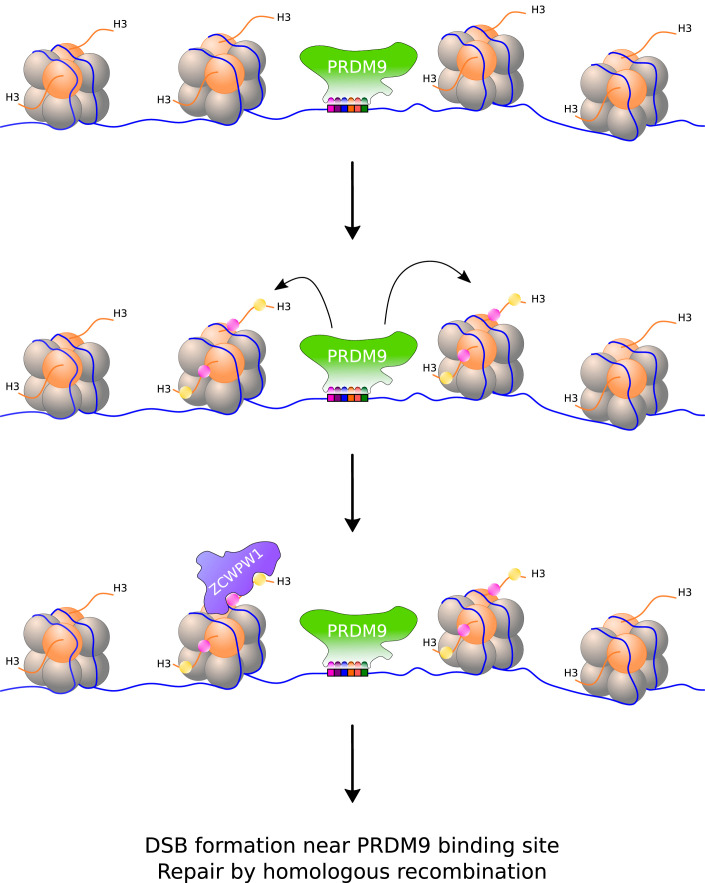
Writing and reading histone modifications that initiate the exchange of genes during meiosis. (Top) Inside cells, DNA (blue line) is stored in repeated units called nucleosomes, which consist of segments of DNA wrapped around a core of eight histone proteins (orange and grey spheres). (Middle) When an enzyme called PRDM9 binds to specific sites in the genome, it modifies the nucleosomes adjacent to it by adding a methyl group to two residues (lysine 4 and lysine 36) on one of the histones (histone 3). These histone modifications are represented as pink and yellow dots, and may be on the same or different proteins, and on one or both PRDM9-flanking nucleosomes. (Bottom) A protein called ZCWPW1 contains two domains that can recognize the modifications deposited by PRDM9. During meiosis, double stranded breaks (DSBs) in DNA form at or around PRDM9 binding sites and are repaired by homologous recombination. ZCWPW1 binding promotes efficient DSB repair via an unknown mechanism.

Now, in eLife, three groups based in China, the United States and United Kingdom report that a protein called ZCWPW1 is able to read the sites where PRDM9 has modified histone H3. Todd Macfarlan, from the Eunice Kennedy Shriver National Institute of Child Health and Human Development, and co-workers showed that ZCWPW1 identifies and binds to these histone marks ‘in vitro’. The team also found ZCWPW1 attached to sites bound by PRDM9 in live spermatocytes – cells that meiotically divide to form sperm – which had been taken from mice. It is common for other enzymes to modify one of the two lysine residues at different sites in the genome. However, ZCWPW1 binds to histone H3 much more strongly when both lysine residues have been modified, allowing it to have a high-level of specificity for the epigenetic marks produced by PRDM9 (Figure 1; Mahgoub et al., 2020).

Another group, led by Hongbin Liu from Shandong University, revealed that ZCWPW1 is present at the same genomic sites as PRDM9, and its localization depends on one of its histone recognition domains (Huang et al., 2020). In addition, Simon Myers and colleagues at the University of Oxford found that when PRDM9 and ZCWPW1 are ectopically expressed in non-meiotic cells, ZCWPW1 still binds to sites in the genome where PRDM9 has deposited a histone mark (Wells et al., 2020). These results suggest that factors that initiate meiosis or homologous recombination are not required for ZCWPW1 to read and bind to these epigenetic modifications.

Although the discovery of ZCWPW1 is important, it also raises questions about the role of this protein. Previous work showed that the histone modifications produced by PRDM9 are required to form double stranded breaks (DSBs) in DNA, which initiate the process of meiotic recombination (Diagouraga et al., 2018). However, two of the groups found that ZCWPW1 is not required for the formation of DNA breaks and instead plays a role in repairing DSBs. Indeed, mice lacking the gene for ZCWPW1 are not able to fully repair meiotic DSBs, resulting in some of the chromosomes in spermatocytes remaining broken. This causes male mice to be sterile, showing that ZCWPW1 is essential for fertility (Huang et al., 2020; Li et al., 2019; Mahgoub et al., 2020; Wells et al., 2020). It is possible that ZCWPW2, a protein highly similar to ZCWPW1, may be involved in DSB formation. However, as several other proteins might also recognize the epigenetic marks deposited by PRDM9, many other scenarios could also be envisioned.

Another question is how does ZCWPW1 promote DNA repair? Given the many molecular steps required for fixing DSBs, several hypotheses can be proposed. For instance, ZCWPW1 might be involved in the early steps of mending these DNA breaks by promoting interactions between homologous chromosomes. Another possibility is that when ZCWPW1 binds to histones it causes chromatin to re-organize, which could initiate several of the mechanisms required for repair. In non-meiotic cells, multiple modifications of chromatin are known to be important for efficiently repairing DSBs (Wilson and Durocher, 2017). Combining mouse genetics with molecular strategies that directly analyze the machinery that fixes breaks in DNA could shed light on some of these questions.

These findings collectively show that PRDM9 and ZCWPW1 work together to recombine DNA during meiosis. This cooperation is also shown by the co-evolution of the genes that encode these two proteins in vertebrates (Mahgoub et al., 2020; Wells et al., 2020). Together, all three studies provide a beautiful example of how a protein that writes marks over the genetic code collaborates with a protein that reads these epigenetic modifications.
